# Yeast ceramide synthases, Lag1 and Lac1, have distinct substrate specificity

**DOI:** 10.1242/jcs.228411

**Published:** 2019-06-24

**Authors:** Márton Megyeri, Rupali Prasad, Giora Volpert, Andrzej Sliwa-Gonzalez, A. Galih Haribowo, Auxiliadora Aguilera-Romero, Howard Riezman, Yves Barral, Anthony H. Futerman, Maya Schuldiner

**Affiliations:** 1 Department of Molecular Genetics, Weizmann Institute of Science, Rehovot 7610001, Israel; 2 Department of Biomolecular Sciences, Weizmann Institute of Science, Rehovot 7610001, Israel; 3 Institute of Biochemistry, Department of Biology, ETH Zürich, Zürich 8093, Switzerland; 4 Department of Biochemistry and NCCR Chemical Biology, University of Geneva, Geneva 1211, Switzerland

**Keywords:** Yeast, *Saccharomyces cerevisiae*, Lag1, Lac1, Phytoceramide, Dihydroceramide, Ceramide synthases, Aging

## Abstract

*LAG1* was the first longevity assurance gene discovered in *Saccharomyces cerevisiae*. The Lag1 protein is a ceramide synthase and its homolog, Lac1, has a similar enzymatic function but no role in aging. Lag1 and Lac1 lie in an enzymatic branch point of the sphingolipid pathway that is interconnected by the activity of the C4 hydroxylase, Sur2. By uncoupling the enzymatic branch point and using lipidomic mass spectrometry, metabolic labeling and *in vitro* assays we show that Lag1 preferentially synthesizes phyto-sphingolipids. Using photo-bleaching experiments we show that Lag1 is uniquely required for the establishment of a lateral diffusion barrier in the nuclear envelope, which depends on phytoceramide. Given the role of this diffusion barrier in the retention of aging factors in the mother cell, we suggest that the different specificities of the two ceramide synthases, and the specific effect of Lag1 on asymmetrical inheritance, may explain why Δ*lag1* cells have an increased lifespan while Δ*lac1* cells do not.

## Abstract (precis)

**Highlighted Article:** Distinct substrate specificities of Lag1 and Lac1, the two yeast ceramide synthases, are revealed, shedding light on their physiological roles.

## INTRODUCTION

The use of model organisms has resulted in important contributions to our understanding of aging. Studies in the yeast *Saccharomyces cerevisiae* (‘yeast’ hereafter) have relied on systematic screens utilizing collections of strains mutated in every yeast gene to uncover those that determine replicative life span (RLS) or the number of cell divisions that each cell can undertake (Mortimer and Johnston, 1959). The first such screen (Egilmez et al., 1989) demonstrated that loss of *LAG1* caused a 50% increase in RLS and thus it was designated as the first longevity assurance gene (LAG) (D'mello et al., 1994). Efforts to understand the effect of Lag1 on aging were complicated by the fact that Lag1 has a biochemical function as a ceramide synthase (CerS), and its close homolog, Lac1 (Jiang et al., 1998), was suggested to have an identical biochemical function (Guillas et al., 2001; Schorling et al., 2001). *LAG1* and *LAC1* are thought to be redundant, because deletion of both genes is lethal, while either of the two can be deleted with no loss of viability and with no major changes in the sphingolipid (SL) content of cells (Barz and Walter, 1999; Jiang et al., 1998). Surprisingly, deletion of *LAC1* has no effect on RLS (Jiang et al., 2004), which suggests that it may have a different role in cell physiology.

Yeast cells divide in an asymmetric manner as budding mother cells increase their replicative age while their daughter cells are born rejuvenated (Barton, 1950). This behavior is also recapitulated in asymmetrically dividing cells of higher eukaryotes such as mammalian stem cells (Denoth-Lippuner et al., 2014a). One of the factors enabling rejuvenation of daughter cells is asymmetric segregation of senescence factors (Denoth-Lippuner et al., 2014a). These factors include extra-chromosomal ribosomal DNA circles (ERCs) (Sinclair and Guarente, 1997), protein aggregates (Saarikangas and Barral, 2015), carbonylated proteins, oxidized lipids, damaged mitochondria (Denoth-Lippuner et al., 2014a) and unfolded proteins in the endoplasmic reticulum (ER) (Clay et al., 2014). In yeast, segregation of senescence factors anchored in membranes, such as unfolded proteins or ERCs attached to the nuclear envelope, relies on a lateral diffusion barrier that restricts the movement of these factors, excluding them from passing from mother to daughter cells (Clay et al., 2014; Denoth-Lippuner et al., 2014b; Saarikangas et al., 2017; Shcheprova et al., 2008). It has recently been shown that segregation of aging factors in neuronal stem cells of rodent brains also requires similar lateral diffusion barriers (Moore et al., 2015).

Essential structural components of diffusion barriers are ceramides and simple sphingolipids (SLs) (Clay et al., 2014). SLs constitute ∼25–30% of membrane lipids and dramatically influence the biophysical properties of membranes (Alonso and Goñi, 2018; van Meer et al., 2008). Based on the critical role of the ceramide synthase Lag1 in aging, the connection of SLs to diffusion barriers and the unexplained different roles played by the iso-enzymes Lag1 and Lac1 in aging, we attempted to provide a mechanistic explanation as to why *LAG1* is a longevity assurance gene whereas *LAC1* is not.

**Fig. 1.**
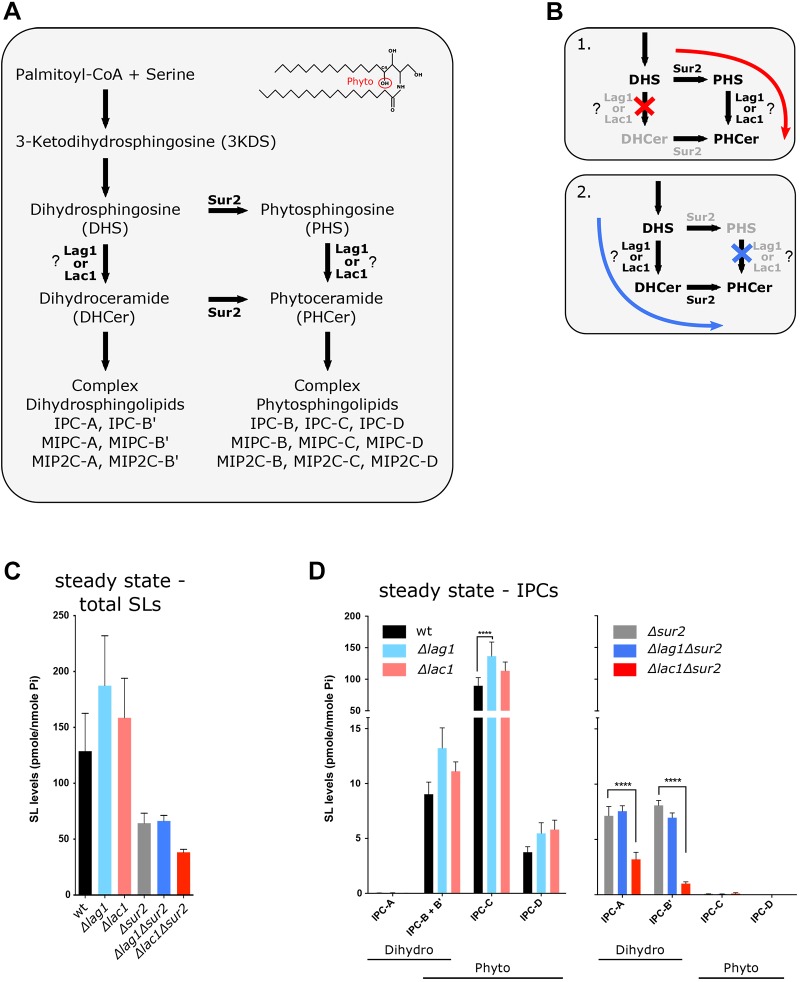
**Effect of deletion of Lag1 and Lac1 on SL levels.** (A) Schematic of the enzymatic steps of the *de novo* SL metabolic pathway in yeast. (B) Lag1 and Lac1 reside at the enzymatic branch point of the SL metabolic pathway created by the C4 hydroxylase Sur2. The enzymatic activity of Sur2 can hinder the study of specificity of Lag1 and Lac1, because deletion of one enzyme can be compensated by the other branch of the pathway (situation 1 and 2). Deletion of Sur2 allows detection of differences in Lag1 and Lac1 specificity. (C) Mass spectrometry (MS) lipidomic analysis of steady-state levels of total SLs. The indicated strains were harvested at logarithmic phase (OD_600_=0.8) and lipids were extracted and analyzed by MS. (D) MS of steady-state levels of inositol-phosphoryl-ceramides (IPCs). IPC-B and IPC-B′ cannot be distinguished when Sur2 is expressed, but in Δ*sur2* background there is no IPC-B. Levels of different SL species are expressed as fmol lipid normalized to the amount of inorganic phosphate determined in the samples (fmol/nmol phosphate) and shown as mean±s.d. from four independent experiments. Statistical significance is indicated (Student's *t*-test, ****P<0.001).

In yeast, the SL metabolic pathway (Megyeri et al., 2016) has two distinct branches: the ‘dihydro’ (DH) and the ‘phyto’ (PH) branches, which differ in their hydroxylation state at C4 of the sphingoid long chain base (LCB) (Fig. 1A). These two forms have different biophysical properties and they perform different cellular roles. For example, phytoceramide is particularly prone to phase separation compared to dihydoceramide (Marquês et al., 2015). Indeed, PH-SLs were proven to be important for diffusion barrier formation (Clay et al., 2014). The two branches are interconnected at two levels of the pathway by the enzymatic activity of the C4 hydroxylase, Sur2 (Haak et al., 1997). Sur2 hydroxylates dihydrosphingosine (DHS) to form phytosphingosine (PHS) and can also convert dihydroceramide (DHCer) to phytoceramide (PHCer) (Haak et al., 1997). The two homologous ceramide synthases, Lag1 and Lac1, lie at the enzymatic branch point in this pathway (Fig. 1A).

In the current study, we set out to examine the LCB specificity of Lag1 and Lac1 to DHS and PHS. We hypothesized that Lag1 preferentially synthesizes PHCer, which has a central role in the diffusion barrier and that this might explain its essential and differential role in longevity. We demonstrate that the bypass pathway created by Sur2 not only renders it difficult to determine such specificity (Fig. 1B) but is also the reason that the different roles of these iso-enzymes have remained convoluted for so long. We therefore uncoupled the enzymatic branch point by means of Sur2 deletion, and used lipidomic mass spectrometry, metabolic labeling and *in vitro* ceramide synthesis assays to determine the enzymatic parameters of the two enzymes. Our data support the notion that Lag1 and Lac1 display different specificity towards DH- or PH-LCBs and that this correlates with their effect on diffusion barrier formation. We suggest that the enzymatic specificity of Lag1 resolves its role in longevity.

## RESULTS

### Unlinking the branch point uncovers differences between Lag1 and Lac1

To investigate the different roles of Lag1 and Lac1, we assayed the effect of single deletions of these iso-enzymes on SL levels. Measuring the steady-state lipid profile of either Δ*lag1* or Δ*lac1* mutants by mass spectrometry (Fig. 1C,D; Fig. S1) revealed that the single mutants do not have a reduction in total SL levels (Fig. 1C). Even when we analyzed SL levels divided according to DH and PH species (Megyeri et al., 2016) (A, dihydro; B′, α-hydroxyl-dihydro; B, phyto; C, α-hydroxyl-phyto and D, α-x-hydroxyl-phyto), we did not detect any major differences between the two mutants (Fig. 1D), in agreement with previous studies (Barz and Walter, 1999; Jiang et al., 1998).

We next deleted *LAG1* and *LAC1* on the background of a Δ*sur2* mutation to see how each synthase loss would be affected in the absence of DHS to PHS conversion, and measured steady-state levels of SLs. As expected, deletion of Sur2 caused a reduction in levels of all PH-SLs (Fig. 1C). However, the double deletion of Sur2 together with Lac1 (Δ*lac1*Δ*sur2*) caused a significant reduction in levels of DH inositol phosphorylceramides (IPC-A and IPC-B′) (Fig. 1D) and all other DH species of more complex SLs (Fig. S1). The fact that loss of Lac1 affects SL synthesis in Δ*sur2* mutants could either suggest that Lac1 is the main enzyme that synthesizes ceramide, rather than Lag1, or that Lag1 has a more specialized role in the PH branch of the pathway.

### Loss of Lac1 but not Lag1 affects strain resistance

**Fig. 2.**
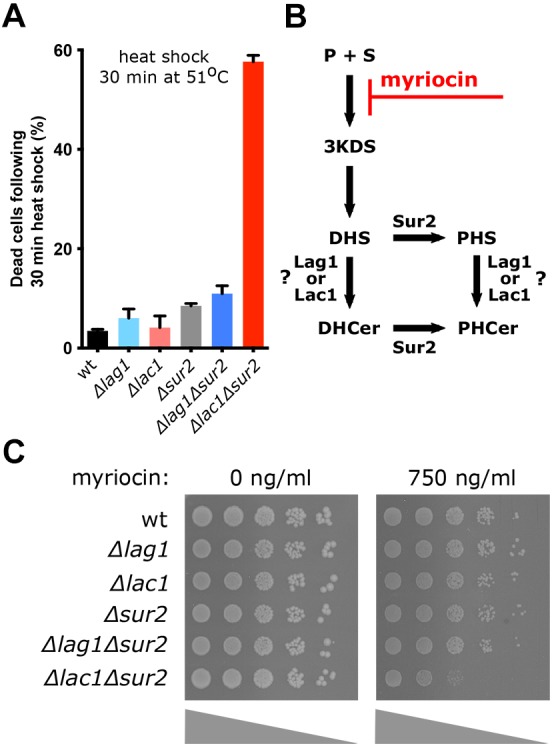
**Biological consequences of uncoupling the SL pathway by deletion of Sur2.** (A) The indicated strains were exposed to heat-shock at 51°C for 30 min. Dead cells were labeled by propidium iodide and analyzed by flow cytometry. The mean±s.d. percentage of dead cells of the whole population are shown. (B) Myriocin is a specific inhibitor of the serine-palmitoyltransferase as indicated on the schematic representation of the initial steps of the SL pathway. (C) Serial dilutions of the indicated strains were spotted on a plate containing no myriocin or 750 ng/ml myriocin and imaged after growth for 24–48 h at 30°C.

The above lipidomic analyses measure lipids at steady state and suggest that Lag1 cannot compensate for the loss of Lac1 in the absence of Sur2. We next challenged cells by heat stress (Dickson et al., 1997; Jenkins et al., 1997), as under these conditions cells require rapid synthesis of SLs to reduce membrane fluidity. We measured the number of dead cells by flow cytometry of propidium iodide-stained cells (Elbaz-Alon et al., 2014) and demonstrated that Δ*lac1*Δ*sur2* cells die at a much higher rate than wild-type cells when subjected to temperatures of 51°C (Fig. 2A) for 30 min, despite no change in viability at normal ambient temperatures (data not shown).

To further characterize whether the effect observed at steady state is a result of decreased flux in the SL biosynthetic pathway rather than an indirect effect, we tested the sensitivity of the strains to the serine palmitoyltransferase (SPT) inhibitor, myriocin (Miyake et al., 1995) (Fig. 2B). Indeed, Δ*lac1*Δ*sur2* cells were more sensitive to myriocin compared to other strains (Fig. 2C), which suggests that in the absence of the PH branch (Δ*sur2*), Lac1 can cope better with lower substrate availability than Lag1.

### *De novo* synthesis of SLs is disrupted in Δ*lac1*Δ*sur2* strains

**Fig. 3.**
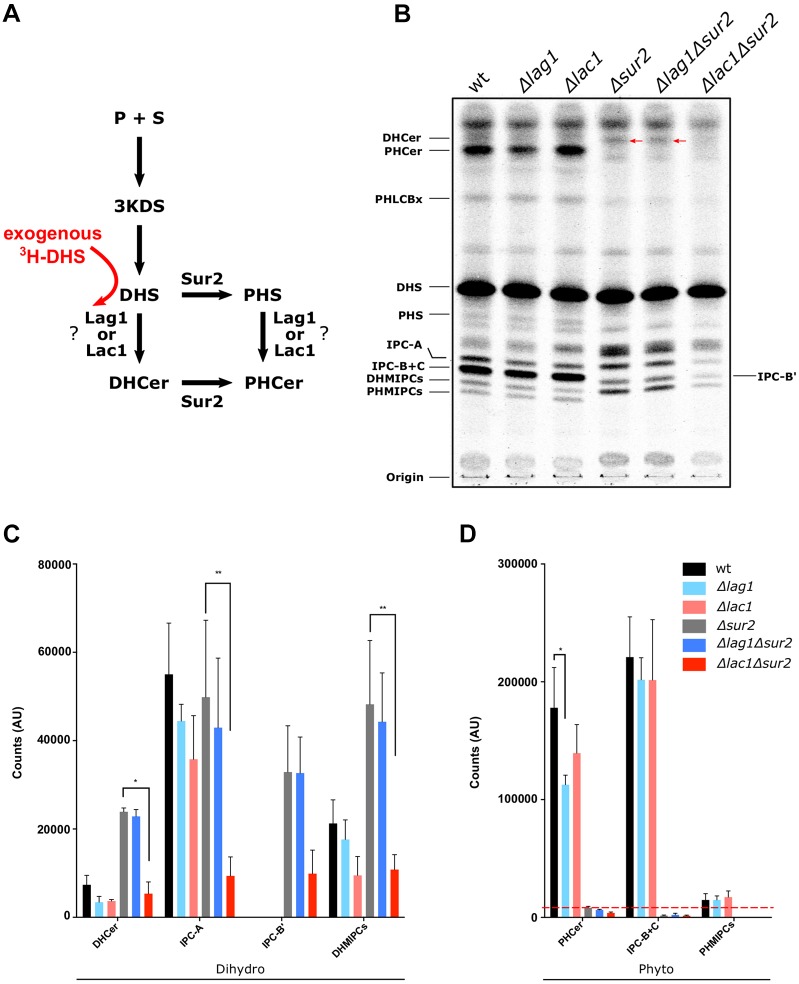
**Metabolic labeling suggests different flux through the SL pathway in Δ*sur2* strains.** (A) Schematic of the initial steps of the *de novo* SL synthesis pathway with the position of exogenously added radiolabeled DHS, to enable metabolic labeling, highlighted in red. (B) The different strains were labeled using 0.8 μCi [4,5 ^3^H]-DHS at 30°C for 30 min. Lipids were extracted and separated by thin layer chromatography (TLC) with chloroform:methanol:2M acetic acid (18:10:2 v/v) as the developing solvent, and then visualized on a phosphor imager. Red arrows denote the DHCer bands, quantified in C. (C,D) The radioactivity for DH-SLs (C) and PH-SLs (D) detected in B were quantified and shown as mean±s.d. as arbitrary units (AU) from three independent experiments. Statistical significance is indicated (Student's *t*-test, *P<0.05, **P<0.01). The dashed red line indicates the detection limit.

Our data in the Δ*sur2* background suggests that Lac1 is the main enzyme that supports the dihydro branch of the SL synthesis pathway. This was tested by metabolic labeling using ^3^H-DHS (Fig. 3A). As expected, deletion of Sur2 resulted in reduced levels of synthesis of all PH-SL species (Fig. 3B,D): PHCer and IPC-B+C were synthesized at very low levels. Moreover, in the Δ*sur2* mutant, we observed increased synthesis of the DHCer species IPC-B′ (Fig. 3B,C), which was almost undetectable in the wild type, suggesting that in the absence of PHCer production, LCBs are channeled into DH species. In agreement with our previous data, the Δ*lac1*Δ*sur2* mutant produced very low levels of SLs (both DHCer and complex SLs) (Fig. 3B,C). In general, metabolic labeling demonstrates bigger differences between the strains than steady-state levels measured by mass spectrometry. This may explain the high sensitivity of Δ*lac1*Δ*sur2* to heat shock or inhibitors when high flux is required in the pathway.

The data presented thus far could not differentiate whether Lag1 cannot support the high demands of SL biosynthesis in the absence of Lac1, or whether Lag1 prefers a different substrate to DHS, such as PHS. However, we noticed that without the deletion of Sur2 when PH-SLs are produced normally, there is a slightly higher production of PHCer in Δ*lac1* mutants compared to Δ*lag1.* While this reduction is quite small it was significant and therefore may suggest that Lag1 has a preference towards PHS as a substrate (Fig. 3D).

### *In vitro* assays demonstrate differential substrate specificity of Lag1 and Lac1

**Fig. 4.**
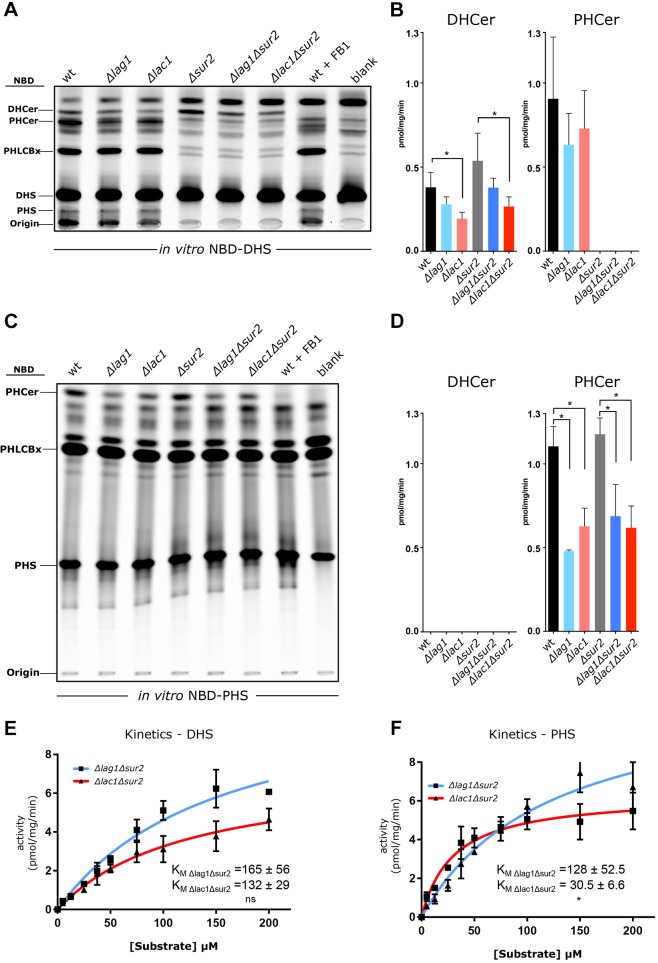
***In vitro* CerS assays suggest differential substrate specificity for Lag1 and Lac1.** (A) Cells were grown until OD_600_=0.3–0.4, harvested, frozen and lysed using a cryo-mill. A 30 μM final concentration of NBD–DHS was added to 0.5 mg of cell lysates from the indicated strains and incubated for 30 min at 37°C. The reaction was stopped by the addition of chloroform:methanol (1:2 v/v). Lipids were extracted and separated by TLC using chloroform:methanol:2M ammonia (40:10:1 v/v) as the developing solvent. The fluorescence intensity of the samples was visualized by a Typhoon FLA 9500 scanner (GE Healthcare). (B) The fluorescence intensity detected in A for DHCer and PHCer were quantified and shown as mean±s.d. in pmol mg^−1^ min^−1^ from three independent experiments. Statistical significance is indicated (Student's *t*-test, *P<0.05). (C) The same cell lysates were used and *in vitro* assay performed as in A, but the substrate was 30 μM NBD–PHS. Lipids were extracted as in A and were separated in solvent system chloroform:methanol:2M acetic acid (18:10:2 v/v) and visualized as in A. (D) The fluorescence intensity detected in C for PHCer was quantified and shown as mean±s.d. from three independent experiments. No fluorescence was detected for DHCer. Statistical significance is indicated (Student's *t*-test, *P<0.05). (E,F) Cells were grown until OD_600_=0.3–0.4 then harvested, frozen and lysed using a cryo-mill. Increasing concentrations of NBD–DHS (E) or NBD–PHS (F) were added to 0.4 mg of cell lysates from the indicated strains and incubated for 30 min at 37°C. The apparent *K*_m_ values of the CerS proteins were determined from three independent experiments and are shown for the indicated strains. Statistical significance is indicated (Student's *t*-test, *P<0.05).

The substrate specificity of Lag1 and Lac1 towards DHS and PHS was measured by an *in vitro* CerS assay. To overcome the low solubility of C26-CoA (the preferred acyl CoA used by yeast CerS), we included human acyl-CoA-binding protein-1 (ACBP1, also known as DBI) in the assay (Ferreira et al., 2017), which was performed using a fluorescent conjugate, nitrobenzoxadiazole (NBD) on the lipid species giving rise to NBD–DHS or NBD–PHS. When using NBD–DHS, we observed that, with or without the Sur2 deletion, the Δ*lac1* strains, where only Lag1 is active, synthesize less DHCer (Fig. 4A,B). However, when using NBD–PHS as substrate, both Δ*lag1* and Δ*lac1* strains synthesized similar levels of PHCer (Fig. 4C,D). Since the same amount of substrate was synthesized when only Lag1 or Lac1 was present in the lysates, and since Lag1 is expressed at lower levels (Fig. S2), it suggests that Lag1 is better than Lac1 in converting PHS to PHCer.

We next determined the *K*_m_ of Lag1 or Lac1 towards DHS and PHS using the Δ*lag1*Δ*sur2* or Δ*lac1*Δ*sur2* mutants, respectively. No significant differences were observed in the *V*_max_ of either strain towards DHS and PHS, although a significant difference in the apparent *K*_m_ of Δ*lag1*Δ*sur2* versus Δ*lac1*Δ*sur2* was observed using PHS, but not DHS (Fig. 4E,F), suggesting that Lag1 (Δ*lac1*Δ*sur2*) has a higher affinity towards PHS than Lac1 (Δ*lag1*Δ*sur2*), consistent with the metabolic labeling data. While this measured difference in affinity is not an absolute preference (Lac1 can still utilize PHS and vice versa), it is of a magnitude that would affect the rate of synthesis in the presence of limiting amounts of substrate (or high amounts of product that would also limit the reaction). The apparent *K*_m_ values measured for Lag1 and Lac1 in this assay were noticeably higher than the apparent *K*_m_ when measuring mammalian CerS. This is likely due to the lower expression of Lag1 and Lac1 and thus the need to use a higher amount of homogenate in the assays, which as a consequence would lower the amount of available free substrates (Tidhar et al., 2015).

### Deletion of Lag1 and Lac1 differentially affects diffusion barriers

We next focused on the diffusion barriers in the ER membrane and nuclear envelope between the mother and daughter cells, as this is a membrane region that is known to affect inheritance of aging factors (Shcheprova et al., 2008). It was previously demonstrated that this barrier requires PH-SLs for its formation (Clay et al., 2014), and we could indeed show that the barrier is lost in a Δ*sur2* background (Fig. S3). This dependence on PH-SLs suggests why Lag1, which demonstrated higher affinity towards PHS, might have a more central role in this path.

**Fig. 5.**
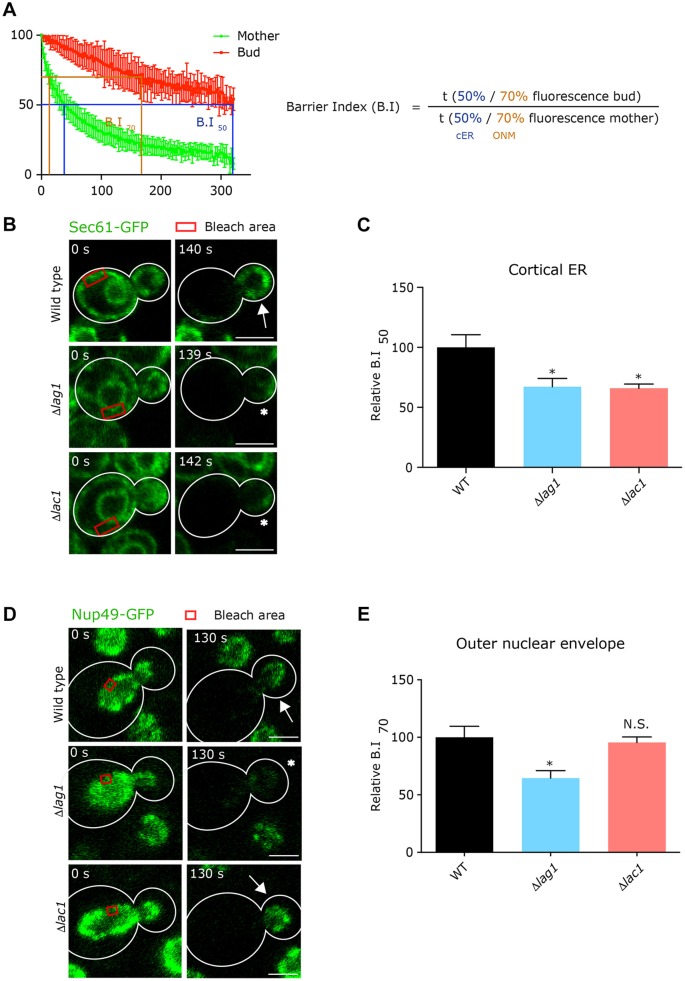
**The diffusion barrier between mother and daughter nuclear envelope is specifically affected by loss of Lag1.** (A) Definition of the barrier index (B.I.) from the kinetics of fluorescence loss. In the case of the cortical ER (cER), B.I. corresponds to the time it takes to lose 50% of the original fluorescence in the bud divided by the time it takes to lose 50% fluorescence in the mother cell (B.I._50_). In the case of the outer nuclear membrane (ONM), the B.I. is calculated the same way as in the cortical ER, but using the time it takes to lose 30% of the original fluorescence in both mother and bud compartments (B.I._70_). (B) Representative images from FLIP experiments on wild-type, Δ*lag1* and Δ*lac1* cells expressing the ER membrane marker, Sec61–GFP. (C) B.I._50_ of the wild-type, Δ*lag1* and Δ*lac1* cells expressing Sec61–GFP. (D) Representative images from FLIP experiments on wild-type, Δ*lag1* and Δ*lac1* cells expressing Nup49–GFP, a marker of the nuclear envelope, during early anaphase. (E) B.I._70_ values for wild-type, Δ*lag1* and *Δlag1* cells expressing Nup49–GFP. Graphs display mean±s.e.m., *n*>40 cells. *P<0.05; N.S., not significant (unpaired *t-*test). Scale bars: 3 μm. Arrows indicate where bud does not and asterisks indicate where bud does lose fluorescence intensity after photobleaching.

The barrier in the cortical ER assembles slowly shortly after the bud starts to emerge from the surface of the mother cell and remains there until cytokinesis (Luedeke et al., 2005). In contrast, the barrier in the nuclear envelope assembles rapidly as the nucleus starts to divide and disappears as the nucleus completes division 10–15 min later (Shcheprova et al., 2008). Therefore, the nuclear barrier is much more transient than the cortical ER and its formation requires rapid build-up of ceramides and specifically PH forms. To follow the diffusion barrier between the mother and daughter either in the cortical ER or in the nuclear envelope (Luedeke et al., 2005; Shcheprova et al., 2008), we used fluorescent loss in photobleaching (FLIP) experiments (Bolognesi et al., 2016; Ellenberg et al., 1997) where the ER is bleached in the mother cell continuously. If the daughter can exchange ER membrane proteins with the mother then its ER will also become rapidly photobleached. Conversely, if there is a diffusion barrier, the daughter ER will remain with high fluorescence (Clay et al., 2014). We used a ‘barrier index’ (B.I.) (Bolognesi et al., 2016) to quantify this phenomenon, defined as the time taken to go down to a certain percent of the original fluorescence in the bud divided by the time it takes to reach a similar loss in the mother (Fig. 5A). A decreased B.I. value indicates that the confinement of photobleached proteins to the mother is less effective, and hence, the barrier is weaker (Fig. 5A).

Using the ER resident protein Sec61–GFP as a reporter for the cortical ER, we observed the presence of a strong lateral diffusion barrier at the bud neck in control cells (Fig. 5B). In contrast, the B.I. to reach 50% (B.I._50_) fluorescence was significantly decreased for the cortical ER barrier in both Δ*lag1* and Δ*lac1* strains (Fig. 5B). To track the nuclear envelope, we used the nuclear pore complex component Nup49–GFP. We found that in the nuclear envelope (Fig. 5C), only the Δ*lag1* strain displayed a significant decrease in the B.I. index [this time using 70% loss of fluorescence (B.I._70_)]. Loss of both Lag1 and Sur2 (Δ*lag1*Δ*sur2*) or Lac1 and Sur2 (Δ*lac1*Δ*sur2*) had no additive effect, supporting our hypothesis that in the complete absence of PHS (in the absence of Sur2) there is no additional importance for which synthase is present (Fig. S3).

## DISCUSSION

In this study we attempted to understand the mechanism by which *LAG1* is a longevity assurance gene while *LAC1* is not. It has long been believed that Lag1 and Lac1 are redundant enzymes both catalyzing the same enzymatic step in the SL pathway. Deletion of either gene caused no detectable reduction in the growth rate of yeast whereas *Δlag1Δlac1* cells displayed growth defects and a significant delay in ER-to-Golgi transport of glycosylphosphatidylinositol (GPI)-anchored proteins (Barz and Walter, 1999; Jiang et al., 1998). Further experiments revealed that the defect in GPI-anchored protein transport is indeed due to a deficiency in ceramide biosynthesis and not due to a general defect in trafficking (Guillas et al., 2001; Schorling et al., 2001).

**Fig. 6.**
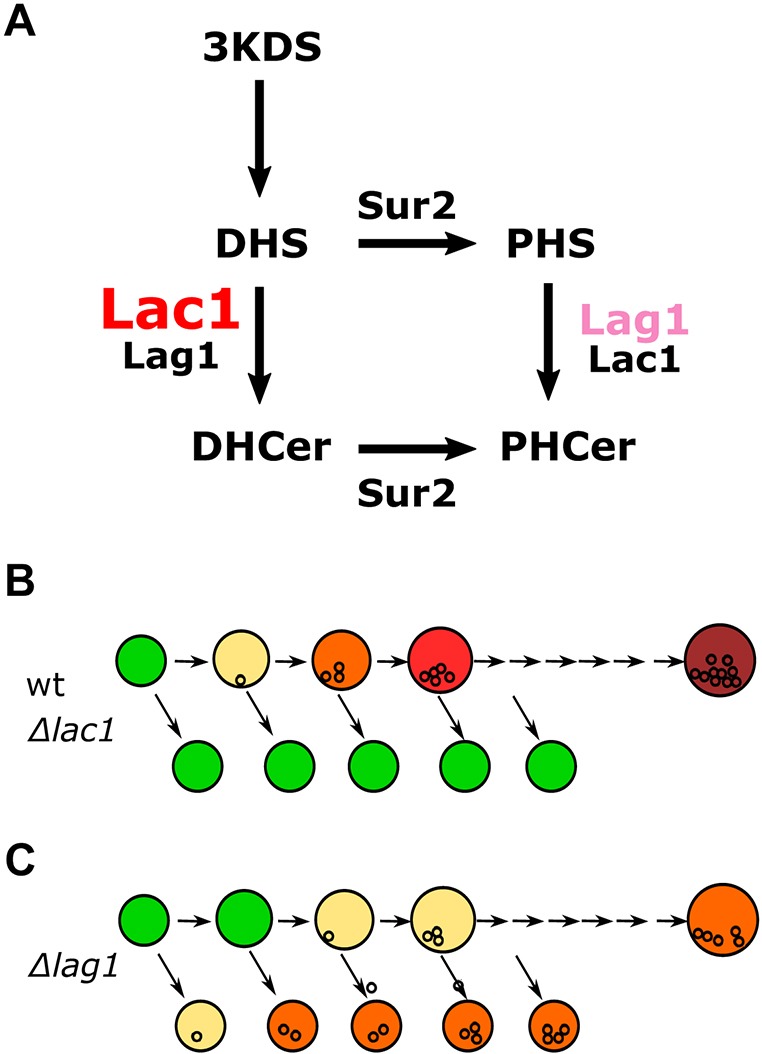
**Reduced diffusion barrier between mother and daughter nuclear envelope in Δ*lag1* strains can explain their longevity phenotype.** (A) Schematic of the increased affinity of Lag1 towards PHS when PHS is in the lower concentration range. Lac1 has high affinity towards DHS in all substrate concentrations. (B,C) Schematic representation of cellular aging in yeast. Mothers actively sequester aging factors and daughter cells do not inherit them. This enables the daughter cells to be born rejuvenated but has a negative effect on the mothers’ fitness. Loss of a diffusion barrier would let aging factors flow into the daughter cells, increasing the mothers’ fitness.

Lag1 and Lac1 lie in the enzymatic branch point of the SL pathway formed by the C4 hydroxylase Sur2 (Megyeri et al., 2016). We now show that the activity of Sur2 hindered determination of the specificity of Lag1 and Lac1 towards the PH- or DH-LCBs. Thus, when we deleted Sur2, we were able to demonstrate that Lag1 and Lac1 have different affinities towards either substrate (Fig. 6A, Fig. 4E,F).

Interestingly, loss of both Sur2 (Clay et al., 2014) and Lag1 affect both the cortical ER and the nuclear envelope diffusion barrier, suggesting that PHCer is required for both. Of note is that steady-state PHCer levels are very similar in both Δ*lag1* and Δ*lac1* mutants, which could be a result of many homeostatic cellular events such as reduction in the breakdown of one form rather than the other (Megyeri et al., 2016). Hence, we suggest that it is the rate of synthesis of PHCer, and not the steady-state levels of PHCer, that determines the capacity to generate the nuclear envelope diffusion barrier. This could be due to the fact that CerS proteins are mainly localized in the nuclear ER (Vallée and Riezman, 2005), and that once formed, PHCer is rapidly transported to the Golgi complex but many other processes could be affecting this and further experimentation is required to better understand this aspect. For example, it would be important to show that PHCer levels are higher in the nuclear ER than the cortical ER, that cortical PHCer levels are high enough to form liquid-ordered domains and that Lag1 preferentially produces the special nuclear pool of PHCer. However, at present our results suggest that Lag1 has an ability to generate PH-SL-rich membranes that serve as a lateral diffusion barrier segregating aging factors in the ER and in the nuclear envelope during asymmetric budding of yeast. Previous studies demonstrated that the barrier in the cortical ER plays a lesser role in the maintenance of aging factors than that of the nuclear envelope (Clay et al., 2014). Since only Δ*lag1* cells have a weaker diffusion barrier in both the cortical ER and nuclear envelope, we suggest that these cells pass more aging factors to their daughters upon budding than Δ*lac1* cells. This would cause Δ*lag1* cells to accumulate fewer senescence factors and age more slowly than wild-type cells, at a cost to their daughters (Fig. 6B,C). The means by which RLS was measured in the original experiments (counting the number of budding events of the mother) established that losing *LAG1* increases the lifespan of the mother cells, supporting this notion. Our observations that Lag1 and Lac1 display distinct substrate preferences and that this leads to distinct effects on diffusion barriers provide a rationale for why only the Δ*lag1* mutation causes an increased replicative lifespan under standard growth conditions.

Differential substrate specificities (or preferences) towards DH and PH species can be seen at different levels of the pathway. For example, in the conversion step of IPC into mannosyl IPC (MIPC), Csh1 and Csg1 (also known as Sur1) (both in complex with Csg2) exhibit similar activity towards IPC-A and IPC-B′ (DH branch) while Csh1 has weaker activity against IPC-B and IPC-C than Csg1 (PH branch) (Uemura et al., 2003). Moreover, the two ceramidases in yeast (Ypc1 and Ydc1) shows differential substrate specificities, such that Ypc1 hydrolyzes PHCer, whereas Ydc1 hydrolyzes DHCer (Mao et al., 2001). Now that it is clear that two different enzymes control the flux of each branch, this may suggest that the cell regulates the relative abundance of each isoform and that the two branches may play different physiological roles beyond the specific role in aging. This may be extremely significant in the context of the fact that 95% of yeast SLs are of the PH form (Ejsing et al., 2009; Megyeri et al., 2016; Savoglidis et al., 2016).

There have been reports of PH-SLs in mammalian cells (Omae et al., 2004); however, they are minor species and may not have similar functions as in yeast. Interestingly, it has recently been shown that asymmetrically dividing neuronal stem cells have a retention of aging factors mediated by a lateral diffusion barrier (Moore et al., 2015). Little is known at present about whether mammalian CerS show a different affinity towards PHS (Tidhar and Futerman, 2013) compared to DHS or whether other differential specificities of mammalian CerS affect the capacity to form these barriers.

More generally, our work demonstrates differential roles for two enzymes previously thought to have an identical function. It may be that other such iso-enzyme pairs have differential substrate affinities that have been hard to uncover as a result of masking by bypass pathways.

## MATERIALS AND METHODS

### Mass spectrometry lipid analysis

Mass spectrometry of SLs was performed as described previously (da Silveira Dos Santos et al., 2014) with minor modifications. Briefly, cells were grown to early exponential phase in rich yeast medium with 2% glucose (YPD) at 30°C, and their metabolism was quenched with trichloroacetic acid (TCA). Lipids were extracted with the extraction solvent [ethanol, water, diethyl ether, pyridine and 4.2 N ammonium hydroxide (15:15:5:1:0.018 v/v)] together with internal standards (7.5 nmol of 17:0/14:1 phosphatidylcholine, 7.5 nmol of 17:0/14:1 phosphatidylethanolamine, 6.0 nmol of 17:0/14:1 phosphatidylinositol, 4.0 nmol of 17:0/14:1 phosphatidylserine, 1.2 nmol of C17:0-ceramide, and 2.0 nmol of C8-glucosylceramide). The extract was then subjected to mild alkaline hydrolysis using monomethylamine reagent [methanol, water, *n*-butanol, and methylamine solution (4:3:1:5 v/v)] followed by salt removal using water-saturated *n*-butanol. The extract was analyzed by ESI-MRM/MS (TSQ Vantage) in positive mode for ceramides and in negative mode for complex SLs. SL levels were normalized to inorganic phosphate.

### Cell viability analysis by flow cytometry

Propidium iodide-stained dead cells were analyzed using a BD LSR II flow cytometer (BD Biosciences) as previously described (Elbaz-Alon et al., 2014).

### Metabolic labeling with radioactive [4,5 ^3^H]-DHS

[4,5 ^3^H]-DHS was prepared as described (Hirschberg et al., 1993) and further purified by thin layer chromatography (TLC) using chloroform:methanol:2 M acetic acid (18:10:2) as developing solvent. The OD of 1 ml cell culture of the different yeast strains in early logarithmic phase was adjusted to 0.3 in synthetic yeast medium with 2% glucose (SD) complete medium and incubated with or without myriocin (1 µg/ml) for 10 min before labeling with 8 µCi of [4,5 ^3^H]-DHS for 30 min. The cells were placed on ice and the reaction was stopped by the addition of 50 µl of NaN_3_ (10% in water). Cell pellets were washed with 1 ml of ice-cold water and lipids were extracted by 500 µl of chloroform-methanol-water (CMW) (10:10:3) by vortexing with 200 µl of glass beads (Zanolari et al., 2000). Cell debris was separated by centrifugation and the organic phase was transferred to a new tube and dried under nitrogen. Dried samples were dissolved in 100 µl of water-saturated *n*-butanol and extracted with 50 µl of water. The aqueous phase was back-extracted twice with 100 µl of water-saturated *n*-butanol. The combined butanol phases were dried under nitrogen flow and dissolved in 30 µl of CMW and then applied to thin-layer chromatography plates (Merck Millipore) and developed in chloroform:methanol:2 M acetic acid (18:10:2) (Reggiori and Conzelmann, 1998). Radiolabeled lipids were exposed to a tritium-sensitive screen (BAS-IP TR 2025 E, GE Healthcare Life Sciences) for 8 h and visualized and quantified on a Bio-Rad phosphor imager.

### Preparation of yeast cell lysates

Yeast cell cultures were grown until an OD of 0.3–0.4 in YPD overnight. Cells were harvested and washed in ice-cold water, and then frozen in liquid nitrogen. Cells were cryo-lysated using a Retsch Mixer Mill MM400 and were kept at −80°C.

### *In vitro* CerS assay

The *in vitro* ceramide synthase assay was performed similarly to Tidhar et al. (2015) with some modifications. Frozen yeast powder was weighed and dissolved in 20 mM HEPES-KOH pH 7.2, 25 mM KCl, 250 mM sucrose and 2 mM MgCl_2_ containing a protease inhibitor cocktail (Sigma-Aldrich). Protein content was determined using BCA reagent (Pierce). Homogenates (0.4 mg protein except for the 30 μM substrate concentration point where 0.5 mg protein was used) were incubated with increasing concentrations of NBD–DHS or NBD–PHS (Avanti Polar Lipids), 20 μM defatted bovine serum albumin (Sigma-Aldrich), 50 μM C26–CoA (Avanti Polar Lipids) and 5 μM human ACBP1 (Ferreira et al., 2017) for 30 min at 37°C. The reaction was stopped by the addition of chloroform:methanol (1:2 v/v). Lipids were extracted and separated by thin layer chromatography using chloroform:methanol:2M ammonia (40:10:1 v/v) as the developing solvent when NBD–DHS was used as a substrate and in chloroform:methanol:2M acetic acid (18:10:2 v/v) when NBD–PHS was used. NBD-labeled lipids were visualized using a Typhoon 9410 variable mode imager and quantified by ImageQuantTL (GE Healthcare). Kinetics parameters were determined using GraphPad Prism software from three independent experiments performed on lysates originating from separate cell cultures.

### Fluorescence loss in photobleaching measurements

FLIP experiments were performed as previously described (Bolognesi et al., 2016; Clay et al., 2014). Briefly, cells were grown at 30°C on YPD for 1–2 days and re-streaked on YPD for 16–18 h prior to imaging. Cells were next immobilized on a 2% agar pad containing non-fluorescent medium. The cells were imaged on a LSM 780 confocal microscope (Carl Zeiss) with a 63×/1.4 NA objective and a multi-array 32PMT GaAsP detector, using 3% of 488 nm argon laser intensity. ZEN 2011 software (Zeiss) was used to control the microscope. Bleaching was applied with 100 iterations at 100% laser power (110 iterations at 80% laser power for Nup49–GFP FLIP experiments). Due to the different lengths of metaphase and anaphase, experiments with Sec61–GFP consisted of 60 time points (4–6 s intervals), while experiments with Nup49–GFP consisted of 40 time points (3–5 s intervals).

Quantification was performed with ImageJ 1.48u (National Institutes of Health). In FLIP experiments, the total integrated fluorescence density of the entire mother and bud compartments, as well as five neighboring control cells (three control cells were used for Nup49–GFP-expressing cells) were quantified. After normalization of the background fluorescence signal, the fluorescence signal of the mother and bud were normalized to the mean of five neighboring control cells and set to 100% at the beginning of the experiment (after discarding the first time-point). The FLIP values for every cell in an experiment were then pooled and analyzed using GraphPad Prism 6 to fit a one-phase decay curve constraining the first bleaching point to 100%. Each experiment was repeated at least four times with a sample size of ≥10 cells. A mean B.I. was calculated from the B.I. of replicated experiments.

## Supplementary Material

Supplementary information
