# Supplementary material for: Yeast ceramide synthases, Lag1 and Lac1, have distinct substrate specificity
Source: J Cell Sci. 2019 Jun 24;132(12):jcs228411. doi: 10.1242/jcs.228411 (PMC6602303; doi:10.1242/jcs.228411)
Supplement: Supplementary information [file joces-132-228411-s1.pdf]

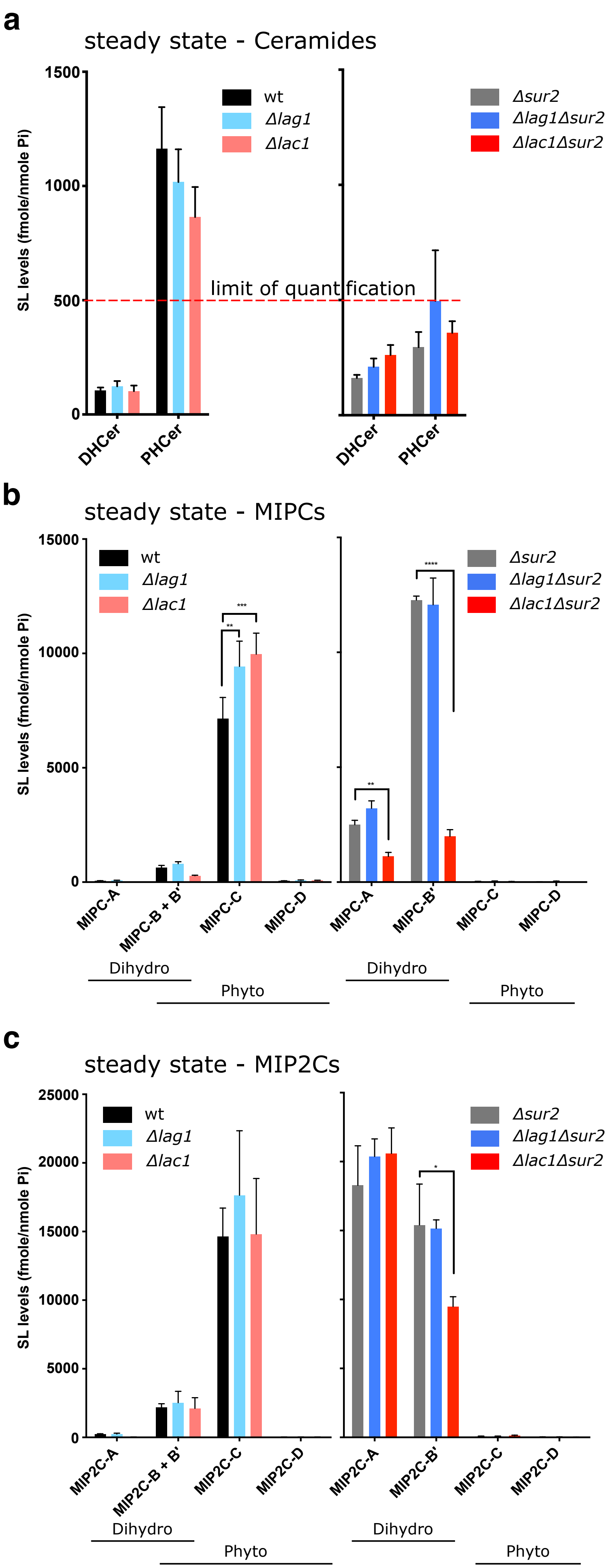

Journal of Cell Science • Supplementary information

Figure S1. Mass spectrometry of steady state levels of SLs.

(a) Mass spectrometry (MS) analysis of steady state levels of ceramides. The indicated strains were harvested at logarithmic phase (OD_{600}=0,8) and lipids were extracted and analyzed by MS. Levels of different species are expressed as fmol lipid normalized to the amount of inorganic phosphate samples (fmol/nmol phosphate) and shown as mean ± s.d from 4 independent experiments. Statistical significance is indicated (Student's t-test, *** p< 0.005, **** p<0.001). (The different B and B' species cannot be distinguished, but in Δ*sur2* background there is no B type of SLs.) The dashed line indicates the limit of quantification of the mass spectrometry method and below this line any measurement could be noise and not a real signal **(b)** MS analysis of steady state levels of mannosyl inositol phosphoryl ceramides (MIPCs). The same biological samples were analyzed as in **a** and results are expressed similarly. **(c)** Steady state levels of mannosyl-diinositol phosphoryl ceramides (MIP2Cs). The same biological samples were analyzed as in **a** and results are expressed similarly.

Journal of Cell Science • Supplementary information

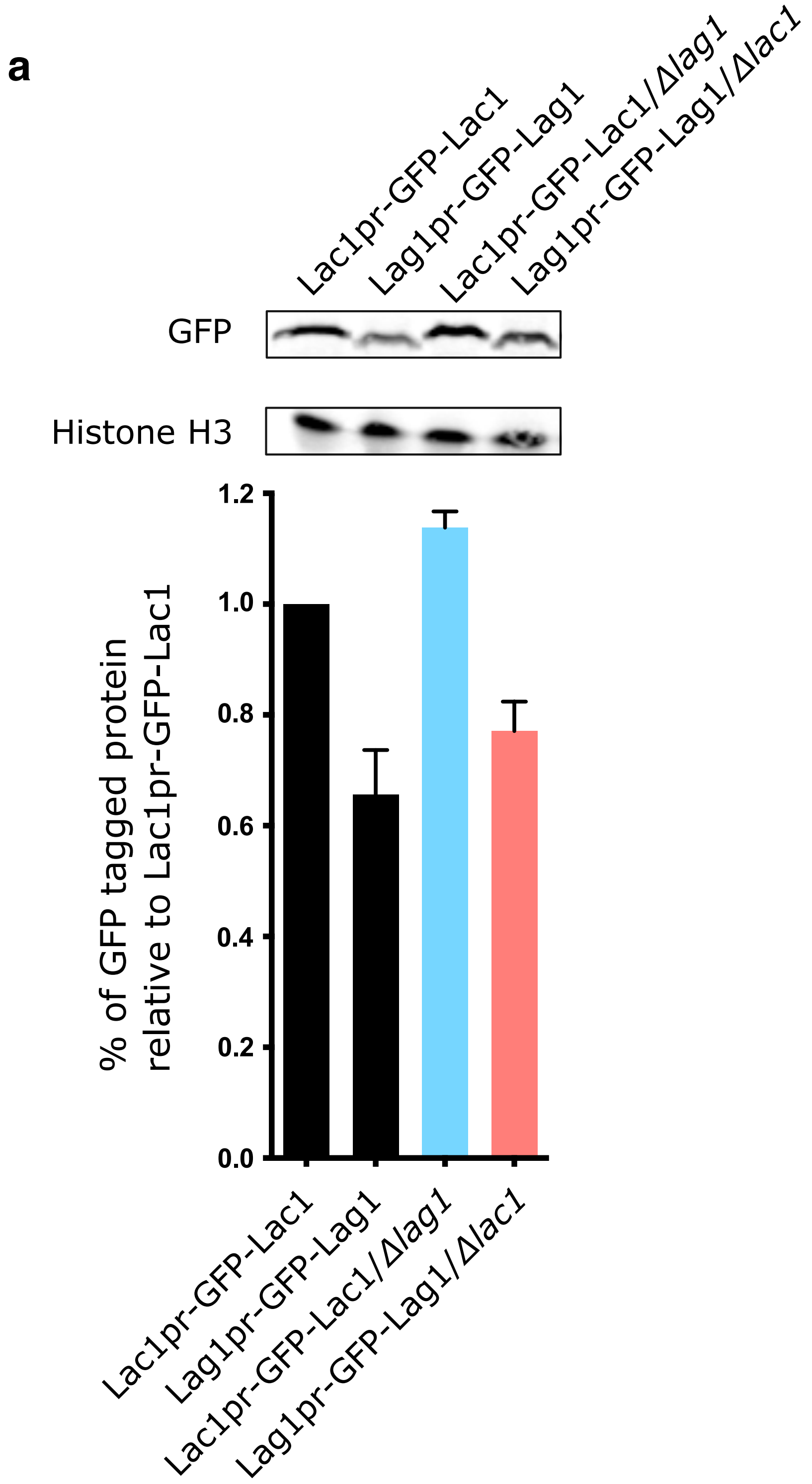

Journal of Cell Science • Supplementary information

Figure S2. Lag1 has lower abundance than Lac1 under normal growth conditions. **(a)** Western blot of whole cell lysates immune-stained against GFP or Histone H3 as a control. Shown are strains that express fusion proteins of Lag1 or Lac1 with GFP at their C'. The integration of the GFP was performed into the endogenous genetic locus and left both genes regulated by their endogenous promoter. Strains were measured either when both enzymes were present or when one of the enzymes was deleted to assay for potential compensatory increase in abundance **(b)** Quantitation of **a** from three independent experiments demonstrating that Lag1 is expressed at lower levels than Lac1 and is not upregulated in response to loss of Lac1.

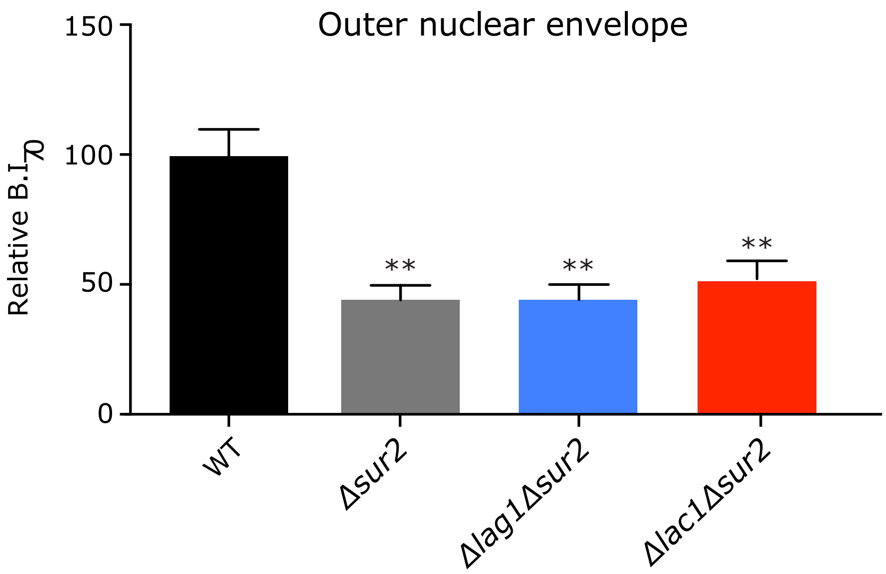
Outer nuclear envelope
Relative B.I %
150
100
50
0
**
**
**
WT
Δsur2
Δlag1Δsur2
Δlac1Δsur2

Figure S3. Deletion of Sur2 affects the diffusion barrier between mother and daughter. The BI70 values for wild type, *Δsur2, Δlag1sur2* and *Δlac1sur2* Nup49-GFP expressing cells are shown here. Graphs display mean ± SEM, n>30 cells. *p<0.05 (unpaired t-test).

Journal of Cell Science • Supplementary information
